# Quantitative Analysis of Small Intestinal Motility in 3D Cine‐MRI Using Centerline‐Aware Motion Estimation

**DOI:** 10.1002/jmri.29571

**Published:** 2024-08-29

**Authors:** Louis D. van Harten, Catharina S. de Jonge, Femke Struik, Jaap Stoker, Ivana Išgum

**Affiliations:** ^1^ Department of Biomedical Engineering and Physics Amsterdam University Medical Center, University of Amsterdam Amsterdam The Netherlands; ^2^ Informatics Institute, University of Amsterdam Amsterdam The Netherlands; ^3^ Department of Radiology and Nuclear Medicine Amsterdam University Medical Center, University of Amsterdam Amsterdam The Netherlands; ^4^ Amsterdam Gastroenterology Endocrinology Metabolism Amsterdam The Netherlands

**Keywords:** dynamic MRI, motility, small intestine, image alignment, Crohn's disease

## Abstract

**Background:**

Currently available tools for noninvasive motility quantification of the small intestine are limited to dynamic 2D MRI scans, which are limited in their ability to differentiate between types of intestinal motility.

**Purpose:**

To develop a method for quantification and characterization of small intestinal motility in 3D, capable of differentiating motile, non‐motile and peristaltic motion patterns.

**Study Type:**

Prospective.

**Subjects:**

Fourteen healthy volunteers (127 small intestinal segments) and 10 patients with Crohn's disease (87 small intestinal segments).

**Field Strength/Sequence:**

3.0 T, 3D balanced fast field echo sequence, 1 volume per second.

**Assessment:**

Using deformable image registration between subsequent volumes, the local velocity within the intestinal lumen was quantified. Average velocity and average absolute velocity along intestinal segments were used with linear classifiers to differentiate motile from non‐motile intestines, as well as erratic motility from peristalsis. The mean absolute velocity of small intestinal content was compared between healthy volunteers and Crohn's disease patients, and the discriminative power of the proposed motility metrics for detecting motility and peristalsis was determined. The consensus of two observers was used as referenced standard.

**Statistical Tests:**

Student's *t*‐test to assess differences between groups; area under the receiver operating characteristic curve (AUC) to assess discriminative ability. *P* < 0.001 was considered significant.

**Results:**

A significant difference in the absolute velocity of intestinal content between Crohn's patients and healthy volunteers was observed (median [IQR] 1.06 [0.61, 1.56] mm/s vs. 1.84 [1.37, 2.43] mm/s), which was consistent with manual reference annotations of motile activity. The proposed method had a strong discriminative performance for detecting non‐motile intestines (AUC 0.97) and discernible peristalsis (AUC 0.81).

**Data Conclusion:**

Analysis of 3D cine‐MRI using centerline‐aware motion estimation has the potential to allow noninvasive characterization of small intestinal motility and peristaltic motion in 3D.

**Evidence Level:**

3

**Technical Efficacy:**

Stage 2

Motility of the human small intestine is under‐studied.^1^ This can be primarily attributed to the practical limitations of currently available conventional analysis tools. The reference standard for small bowel motion patterns is manometry,^2^, ^3^ which can yield a detailed and interpretable measurement of motility in the small intestine. However, manometry is used only for measuring the proximal small intestine in general, as the mechanical difficulties of intubating the small intestine hamper more distal measurements. Additionally, due to the invasive nature of manometric measurements, population‐based studies on patterns and differences of small‐intestine motility among healthy volunteers are generally considered unfeasible. This in turn limits the diagnostic value of manometric measurements, as there is little baseline data with which clinical standards can be reliably established. Scintigraphy^4^ and capsule endoscopy^5^ can evaluate the distal small bowel, but their value in evaluating detailed motion patterns of the small bowel is limited.

In recent years, cine‐MRI (also known as dynamic MRI) has emerged as an alternative for evaluating motility in the small intestine.^6^, ^7^ This technique has the potential to quantify motility in the entire small intestine simultaneously with negligible patient discomfort, which implies potential use for screening, low‐barrier clinical use and population‐based studies. However, cine‐MRI is currently not widely used for intestinal motility measurements, as there are two important unfulfilled requirements: the availability of suitable imaging protocols and automatic analysis tools.

Manual motility analysis on cine‐MRI is possible by measuring the in‐plane diameter changes of an imaged segment of intestine,^8^ but this is only viable if there is a specific region of clinical interest, for example near a surgical site in post‐operative imaging. Otherwise, the amount of information present in the images is too large for manual analysis to be clinically feasible. Additionally, when using 2D cine‐MRI, such measurements are only possible if the imaged segment‐of‐interest is perfectly aligned with the acquired imaging plane and no out‐of‐plane motion is present.

While 2D cine‐MRI methods^9^, ^10^, ^11^, ^12^, ^13^, ^14^ can be used to quantify the amount of motion in the intestinal region, a number of important motion characteristics are not visible in 2D MRI scans. Small intestinal motility is a combination of coordinated contractions resulting in antegrade and retrograde peristaltic motion, as well as contractions that result in mixing motion.^15^, ^16^, ^17^ The local magnitude of the motion is not sufficient to differentiate the two; they can only be distinguished by correlating motion at multiple locations with known spacing.^18^ This typically cannot be achieved for the small intestine by acquiring and analyzing 2D MRI data, as it requires the center of a segment of intestine to be and stay within the imaged 2D plane for the duration of the scan.

We propose that estimating the small intestinal motion in 3D cine‐MRI using a centerline‐aware approach may address the limitations of 2D small intestinal motility analysis and enable characterization of different types of motility. By relating motion to intestinal centerlines, the geometry of the intestine is known, enabling evaluation of the coordination of motion between nearby locations. Unlike with existing 2D tools, this approach may allow differentiation between coherent peristaltic motion and incoherent mixing motion and makes small intestinal motility quantification robust to out‐of‐plane motion.

Thus, the aim of this study was to develop a technique to quantify small intestinal motility in 3D cine‐MRI using centerline‐aware motion estimation, capable of differentiating motile, non‐motile and peristaltic motion patterns.

## Materials and Methods

### Dataset Details

The method was developed using data from the Amsterdam UMC, location University of Amsterdam, The Netherlands. Permission for the use of the data was granted by the Medical Ethics Committee of the Amsterdam UMC (NL54884.018.15, NL76851.018.21) and informed consent was obtained from all subjects. The dataset consisted of abdominal 3D cine‐MRI breath‐hold scans from 14 healthy adult volunteers and 10 adult Crohn's disease patients. The included patients were 18 year or older and had endoscopic or histological confirmed Crohn's Disease, one or more small bowel stricture(s) confirmed on endoscopy and/or cross‐sectional imaging and were scheduled for stricture surgery.

### Image Acquisition

Scans were acquired using a 3 T Philips Ingenia MRI scanner (Philips Medical Systems, Best, the Netherlands) with subjects in supine position using a combination of a posterior coil located in the table and an anterior torso coil covering the abdominal region. A coronal 3D dynamic balanced fast field echo (bFFE) sequence was acquired during a breath‐hold period of 18 seconds. The scan parameters were: TE/TR: 1.29/2.6 msec, flip angle: 20°, field of view: 400 × 400 × 35 mm (FH × LR × AP), no gaps, spatial resolution: 1.4 × 1.4 × 2.5 mm, SENSE: 3.5 (RL) × 1 (AP), Half Scan (partial Fourier): *Y* = 1, *Z* = 0.8, resulting in a temporal resolution of one 3D volume (35 mm thickness) per second. The field of view covered only part of the total abdominal volume, but contained the terminal ileum for all subjects. Before scanning, subjects fasted at least 4 hours before ingesting 1000–1600 mL 1.9% mannitol solution (i.e., routine clinical preparation^19^) to increase contrast and distention of the small intestinal lumen. Subjects scanned in the morning, including all volunteers, fasted overnight. The first two volumes in each scan were discarded, as these contained contrast artifacts.

### Reference Standard

For the purposes of this study, a definition of small intestinal segments was necessary, as the acquired field of view of the scans was not large enough to encompass the entire small intestine. The segments were defined as starting and ending at the points where the small intestine entered and left the field of view. We used an existing method for intestinal centerline extraction based on convolutional neural networks (CNNs) to automatically extract small intestinal segments in the images.^20^ Segments were generated from 333 manually placed markers across both datasets (206 in the set of healthy volunteers, 127 in the set of Crohn's disease patients). To ensure only correctly extracted centerlines were included in the evaluation of the presented method, manual quality control was performed on each segment by two raters of 3D cine MRI. The raters were a gastrointestinal (GI) motility MR researcher (CSJ; 9 years of experience) and an abdominal radiologist (FS; 5 years of experience) who were blinded to the quantitative results from the method. Centerline segments were considered failed extractions if less than 75% of the centerline correctly followed the small intestine (i.e., centerlines that crossed the intestinal wall, usually into an adjacent intestinal loop). All failed extractions were excluded from further analysis.

To define the reference labels for each segment, both raters independently evaluated the motility in each intestinal segment. After evaluating the correctness of the automatically extracted centerlines, the raters evaluated the presence of motility on a three‐point scale: motile, <50% motile, or not motile. For each motile segment, the raters additionally evaluated the dominant direction of peristalsis as forward, backward, or bidirectional/mixing motion. Note that our 3D scans (thickness 3.5 cm) did not encompass the complete abdomen and therefore forward and backward did not necessarily refer to propulsion and retropulsion respectively. The centerlines were oriented arbitrarily with respect to the proximal and distal directions. The centerlines typically started and ended at the boundaries of the field of view, meaning it was impossible to determine the physical orientation in our 3D scans. Hence, forward peristalsis denoted movement from the beginning to the end of the centerline, whereas backward peristalsis denoted movement in the opposite direction. After both raters had evaluated all segments separately and blindly, a consensus was established in an additional reading session, where the raters could discuss the classification for each segment. During the reading sessions, the raters determined that there was a lot of ambiguity in the definition of the classes “<50% motile” and “no motility,” with no clear threshold for the amount of movement that should push a mostly non‐motile segment into the “<50% motile” class. Therefore, these classes were merged for the purposes of evaluating the automatic classification. The full annotation protocol as available to the raters can be found in the Appendix.

All unique segments longer than 4 cm that neither rater marked as a failed extraction were included in the analysis. For each pair of non‐unique segments, the shorter segment was excluded.

### Motion Quantification Methods

**FIGURE 1:**
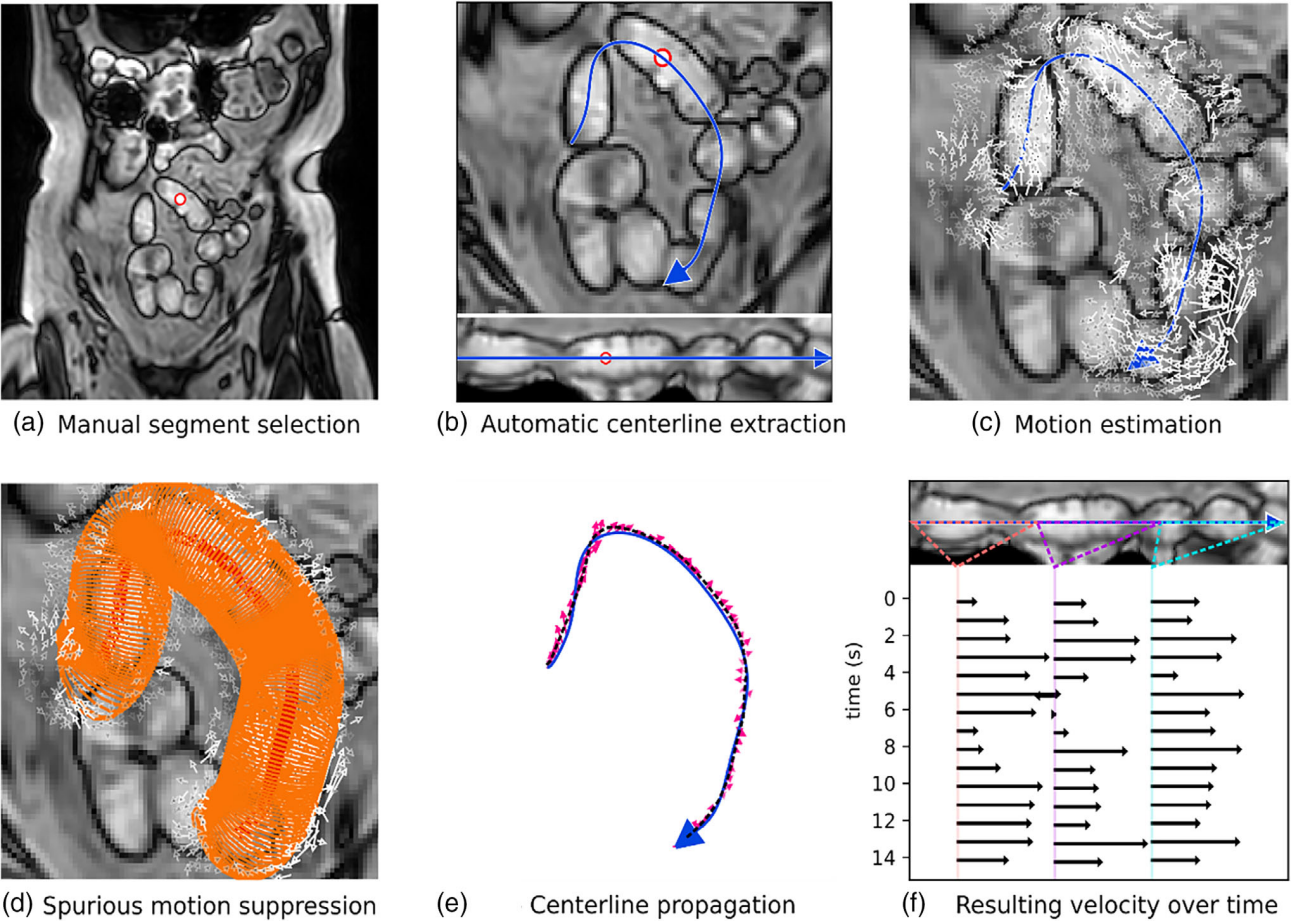
Motility quantification overview. Input to the method is the centerline of a small intestinal segment, which is extracted using an automatic method (a–b). (**a**) The segment‐of‐interest is manually selected by the user (red circle). (**b**) The segment is automatically extracted (blue arrow). Top: projected 2D view in coronal plane, bottom: untangled representation of segment‐of‐interest. (**c**) The motion in the region near the extracted centerline is estimated using deformable image registration, visualized by white arrows; (**d**) the resulting vector field is sampled with spurious motion suppression (red and orange rings). (**e**) The extracted centerline is propagated to the next time point (dashed black line) based on a moving average of the estimated motion (pink arrows). Steps c, d and e are repeated for every time point in the scan. (**f**) The resulting estimated velocities are aggregated along the centerline and over time. For visualization purposes, we average the motion for the beginning of the segment (orange), the middle of the segment (purple) and the end of the segment (teal) at each time point. In the shown example, there is peristaltic motion in the same direction as the centerline direction in all three parts of the segment, resulting in positive velocities (black arrows).

A method was developed that quantified motion in the small intestine. Given a small intestinal segment centerline, the method describes the local velocity within the small intestinal lumen using deformable image registration. An overview of the method is shown in Fig. 1. The resulting motion estimates were used to classify the presence and type of motility using linear classifiers. The method components are described in detail below.

### Motion Estimation

To estimate the motion in the intestinal segments, a deformable registration method was employed that uses cycle‐consistent implicit neural representations,^21^ which are neural networks that represent the motion field between two time points. The method optimized two sinusoidal representation networks (SIRENs^22^) to map the spatial coordinates in one time point to corresponding coordinates in another time point and vice‐versa, resulting in a bidirectional transformation function. This is an extension of the implicit deformable image registration‐method,^23^ leveraging the inverse functions to yield more stable and more accurate registration results.^21^ All neural representations were optimized using an optimization schedule of 1500 iterations and a one‐cycle learning rate policy^24^ to optimize the representation networks, using the normalized cross‐correlation (NCC) with a symmetric Jacobian determinant penalty^21^ and a cycle‐consistency penalty^21^, ^25^ as the objective function.

For the registration process, a tube with a diameter of 40 mm was defined around the segment centerline as the volume of interest. This tube size was chosen since it is larger than any healthy or mildly distended small intestine (i.e., 20–30 mm^1^). Within this volume, the motion field between subsequent time points was estimated. The radius for this volume of interest could be manually expanded by the user for more severely distended intestinal segments, but this was not required for any of the intestinal segments analyzed in this study. The result is a motion vector field around the centerline that can be sampled at an arbitrary resolution, as visualized in Fig. 1c.

### Suppressing Spurious Motion

The motion in the intestines is a combination of intestinal motility and spurious motion, which is motion unrelated to the motility in the intestinal segment‐of‐interest, such as breathing motion, cardiac motion, or motion caused by motility in adjacent intestine segments. To prevent this spurious motion from affecting the motility metrics, spurious motion suppression was employed.

The approach used relied on the intuition that the majority of spurious motion affects both the segment‐of‐interest and the surrounding tissues, while the intestinal motility primarily affects the intestine itself. Hence, a differential sampling method (reminiscent of common‐mode rejection in electronics) was employed: the integral of the motion was evaluated in a large circular region of interest (ROI) around each point on the centerline (in this study a diameter of 30 mm was used, i.e., greater than the small intestine diameter and contained in the 40 mm region for which motion was estimated, as shown in orange in Fig. 1d), which was subtracted from the integral of the motion sampled from a circle with a small radius around each centerline point (with a diameter of 0.5 mm, i.e., much smaller than the small intestine diameter, as shown in red in Fig. 1d). The result is a motion description of just the local motion within the segment‐of‐interest, suppressing the common motion component that is also present in the surrounding tissues. For more severely distended intestines, the diameter of the outer ring could be increased from 30 mm to ensure it falls outside of the intestinal lumen, but this was not required for any of the segments in our dataset.

### Centerline Propagation

To find the segment‐of‐interest centerlines, an automatic centerline extraction method was used, which produced a segment centerline for one time point. However, the segment‐of‐interest may have moved over the course of the cine‐MRI acquisition, both due to breathing motion and due to being pushed around by contractions in either the segment‐of‐interest or in the surrounding intestines. This means that the centerline must be propagated to all available time points. However, reusing the previously computed vector fields between subsequent time points would introduce accumulating registration artifacts from repeatedly moving the centerline, magnifying any minor inaccuracies. Therefore, in this study, each time point volume was also registered directly to the time point volume in which the centerline was defined, resulting in a more accurate propagation result.

The resulting vector fields were sampled at the centerline coordinates and the sampled deformation vectors were smoothed with a moving average window of 8 mm along the centerline. This resulted in a smooth centerline estimate at each time point. Additionally, a moving average window of 3 seconds was applied along the time dimension to encourage temporal smoothness. One such propagation is visualized in Fig. 1e.

### Motility Extraction and Aggregation

To construct the local motility estimates, the sampled motion vectors were evaluated along the intestinal segment centerline. A dot product with the corresponding tangent of the centerline was applied to each sampled motion vector to extract the motion component that was aligned with the centerline, resulting in a quantity that can be interpreted as the local velocity of the intestinal content along the direction of the intestine, expressed in millimeters per second. Note that this velocity can be negative, if the motion vector pointed in the opposite direction compared to the centerline. This process was repeated for each subsequent time point in the cine‐MRI scan to find the local velocities along the centerline over time. For visualization purposes, these velocities were aggregated into an average for the beginning, middle and end of the segment (as shown in Fig. 1f).

From the local velocities, two global metrics were derived that were used to classify the intestinal segments: the mean velocity and the mean absolute velocity. The mean velocity is the mean over both the time and the centerline dimensions, resulting in a measure of average peristaltic motion in the segment. For the mean absolute velocity, the absolute values for all local velocities were taken prior to averaging, resulting in a measure of motility irrespective of directional consistency, both over time and along the evaluated segment. The key difference between these two metrics is that for the mean velocity, opposing motion vectors cancel each other out, whereas this does not happen for the mean absolute velocity. This means that the mean velocity is only large for segments where the motion vectors consistently pointed in the same direction, whereas the mean absolute velocity is large for any segment with large motion vectors, including uncoordinated motion.

### Automatic Motion Classification

Two linear, one‐dimensional classifiers were used to determine the presence of motility and the presence of peristaltic motion. The first classifier used a threshold on the mean absolute velocity in each segment to determine the presence or absence of motility. The second classifier used the mean velocity in each segment to determine the presence and direction of peristaltic motion; the second classifier was only applied to segments which were classified as motile by the first classifier. The thresholds for both classifiers were determined using five‐fold cross‐validation. K‐fold cross‐validation is a resampling procedure used to evaluate machine learning models; here five folds were used. The dataset was split by subject, with all segments from the same subject being grouped in the same cross‐validation fold.

The full automatic analysis was performed on a small GPU cluster containing 8 RTX 2080TI GPUs and an AMD 7401P CPU, clocked at 3 GHz and the computation time was evaluated for each of the components.

### Statistical Analysis

The discriminative power of the proposed motility metrics was evaluated in terms of area under the receiver operating characteristic (ROC) curve (AUC) of each classifier. The automatic classification results were evaluated using accuracy and Cohen's kappa coefficient with the reference annotations. These results were compared with the inter‐rater variability using the same metric. The amount of motility in the healthy volunteers was also compared with the amount of motility in the Crohn's disease patients using a Student's *t*‐test. A *P*‐value <0.001 was considered significant.

## Results

### Descriptive Statistics

A total of 333 segment centerlines were generated. Of these, 41 centerlines were excluded based on length, 9 centerlines were excluded as duplicates and 69 centerlines were marked as extraction failures by at least one of the raters. Finally, a set of 214 unique segments (127 from healthy subjects, 87 from Crohn's disease patients, average segment length 8.5 ± 4.9 cm) was included for the analysis. The absence of motility was significantly more common (34%, 30/87) in the set of Crohn's disease patients, although it also occurred (11%, 14/127) in the set of healthy volunteers. Discernible peristalsis was significantly more common (35%, 44/127) for segments from healthy volunteers, but was also present (11%, 10/87) in segments from Crohn's disease patients.

### Motion Classification

**FIGURE 2:**
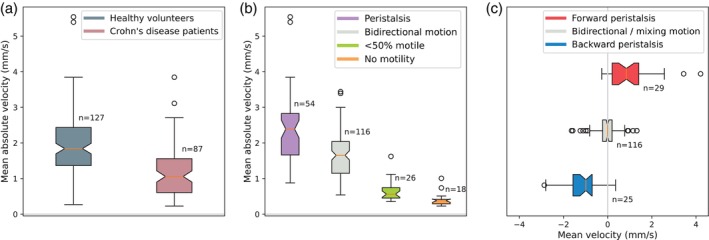
The mean absolute deformation velocity (reflecting magnitude of motility) in each centerline segment, split by dataset (**a**) and by reader consensus class (**b**), and the mean deformation velocity (reflecting directionality of motility) for all motile segments, split by reader consensus peristalsis characteristics (**c**). Annotations show the number of small intestine segments in each distribution.

The mean absolute velocity (reflecting magnitude of motility) was significantly higher for segments in the set of healthy volunteers than the mean absolute velocity for segments in the set of Crohn's disease patients. The mean absolute velocity was also significantly different for motile segments compared to <50% motile and non‐motile segments (Fig. 2a,b; Table 1). The mean intestinal velocity (reflecting directionality of motility) was significantly different for motile segments with forward peristalsis, backwards peristalsis, and bidirectional motion (Fig. 2c; Table 2).

**TABLE 1:** Mean Absolute Velocity of Intestinal Segments (Reflecting Magnitude of Motility), Split by Dataset and by Reference Motility Characteristics

		Median [IQR]
Dataset	Healthy volunteers (N = 127)	1.84 [1.37, 2.43]
	Crohn's disease (N = 87)	1.06 [0.61, 1.56]
Motile segments	All (N = 170)	1.75 [1.33, 2.33]
	Peristalsis present (N = 54)	2.39 [1.66, 2.83]
	Bidirectional motion (N = 116)	1.65 [1.15, 2.04]
Non‐motile segments	All (N = 44)	0.47 [0.38, 0.63]
	<50% motility (N = 26)	0.56 [0.45, 0.74]
	No motility (N = 18)	0.38 [0.29, 0.41]

**TABLE 2:** Mean Velocity of Motile Segments (Reflecting Directionality of Motility), Split by Reference Peristaltic Characteristics

	Median [IQR]
Backward peristalsis (N = 25)	−0.99 [−1.57, −0.70]
Bidirectional motion (N = 116)	0.00 [−0.23, 0.20]
Forward peristalsis (N = 29)	0.85 [0.20, 1.41]

**FIGURE 3:**
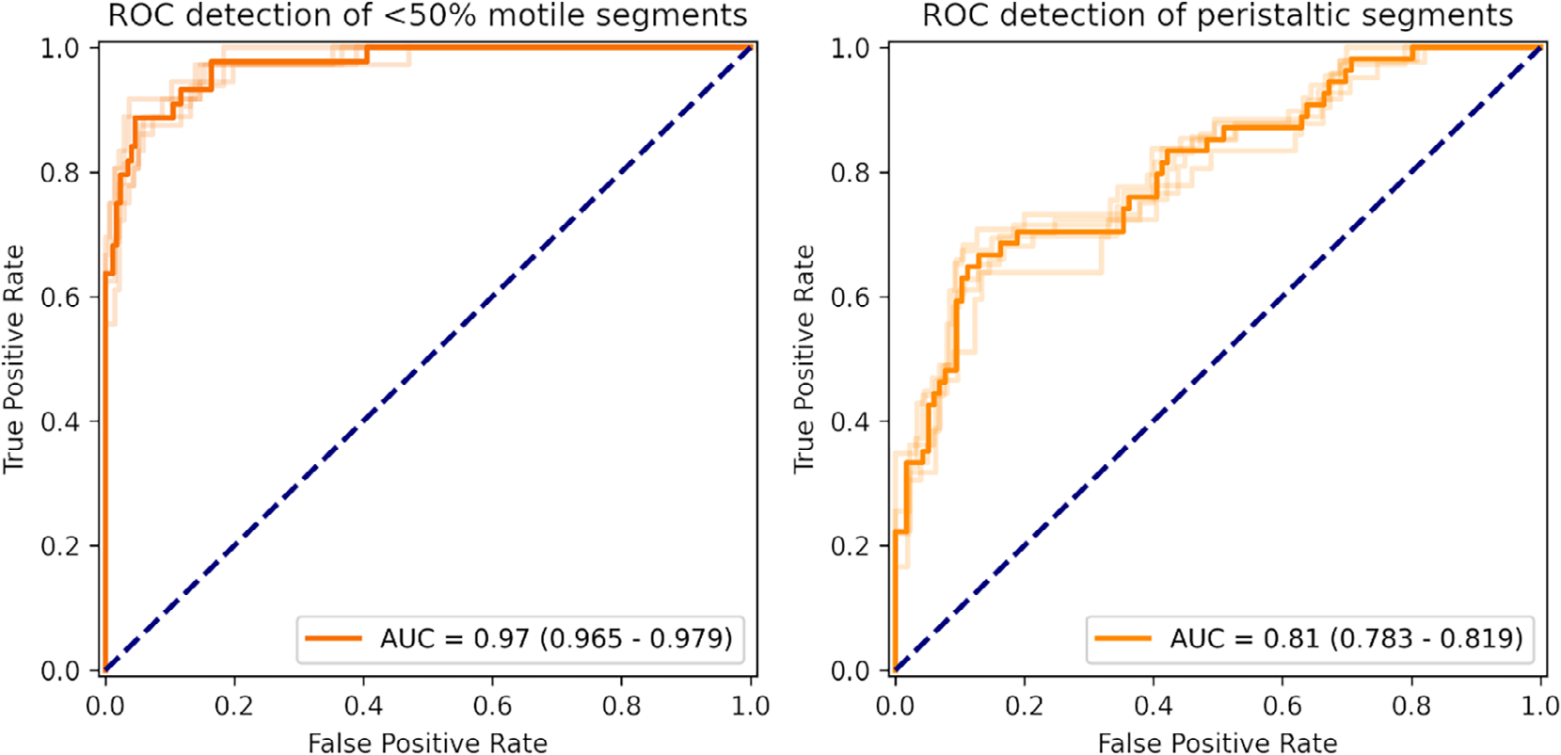
ROC curves for the detection of <50% motile segments (including non‐motile) and the detection of peristaltic motion. Results plotted for all 5 cross‐validation folds with low opacity, average shown as opaque. AUC reported for the mean result, with minimum and maximum values among folds in parentheses.

The receiver operating characteristic (ROC) curves for the detection of <50% motile segments and the detection of peristalsis for each cross‐validation fold are shown in Fig. 3. The mean (range) AUC across all folds was 0.97 (0.975–0.979) for the detection of <50% motile segments and an AUC of 0.81 (0.783–0.819) for the detection of peristalsis.

**FIGURE 4:**
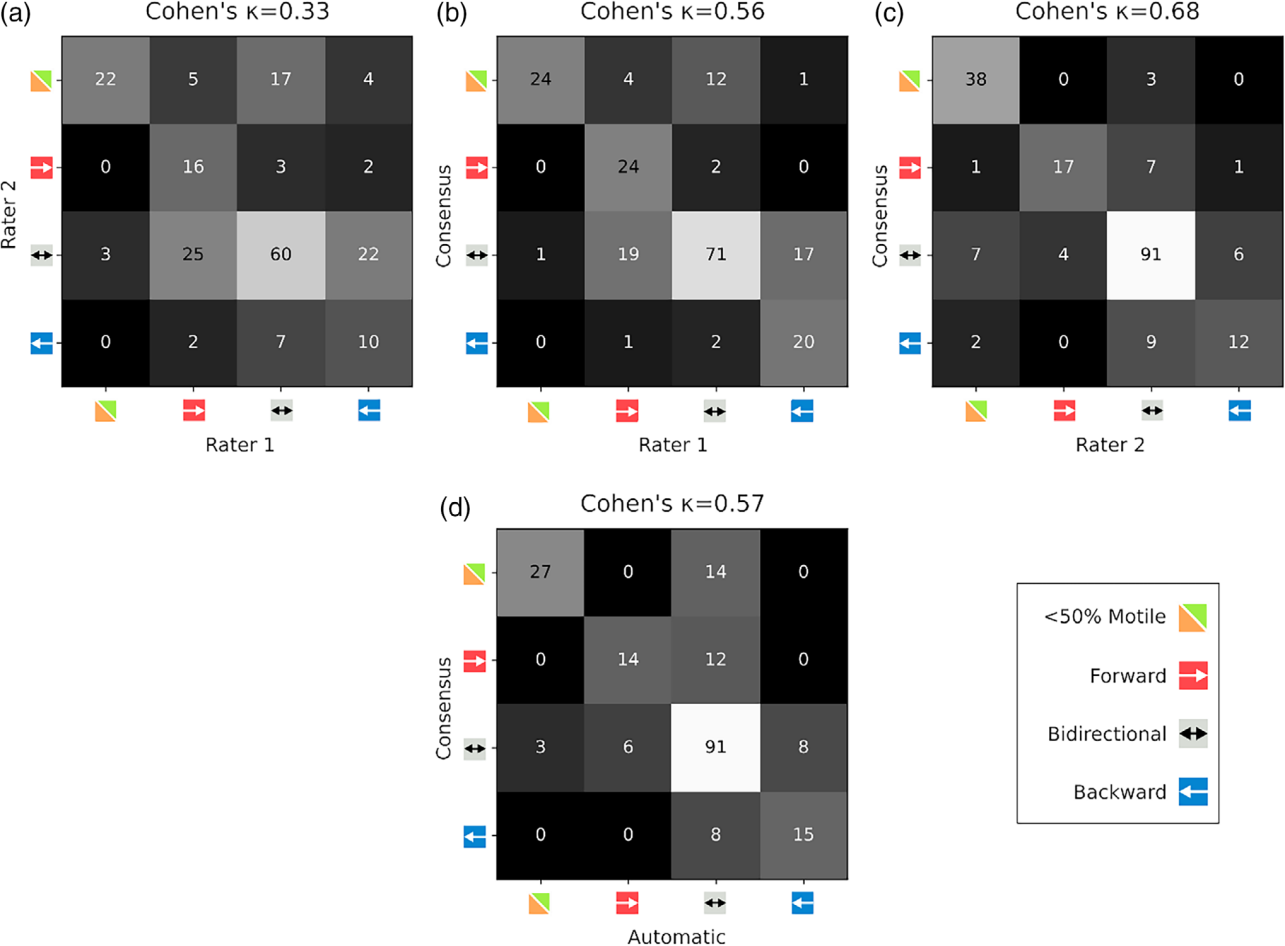
Contingency matrices for the full classification task. Top row: interrater results, comparing both raters against each other (**a**) and both raters against their collective consensus results (**b**, **c**). Bottom row: automatic results compared against the consensus (reference) results (**d**). Thresholds for automatic results obtained from 5‐fold cross‐validation.

Figure 4 shows the contingency matrices comparing the automatic results from the linear classifiers and the inter‐rater results. This shows that the agreement of the automatic classifiers with the manually scored consensus reference was substantially higher (Fig. 4d) than the inter‐rater agreement (Fig. 4a, Cohen's unweighted kappa of 0.57 compared to 0.33). Additionally, it shows that the automatic results had similar agreement with the reference (Fig. 4d) compared to the human raters (Fig. 4b,c): unweighted kappa of 0.57 compared to 0.56 and 0.68 for rater 1 and rater 2, and an accuracy of 68.7% for the automatic method compared to 65.0% and 73.8% for the raters, respectively.

### Motion Patterns

**FIGURE 5:**
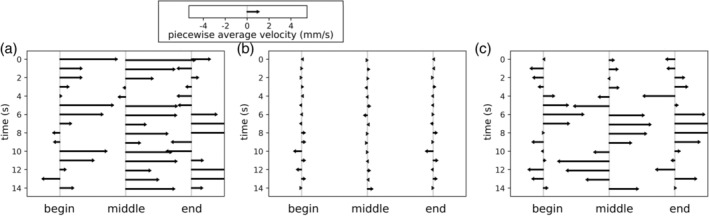
Estimated velocity results along segment centerlines (*x*‐axis), over time (*y*‐axis) for three intestinal segments representing a motile segment (**a**), non‐motile segment (**b**), and a segment with bidirectional motility (**c**) respectively. The arrows along the *x*‐axes indicate the average motion in the beginning, middle and end parts of each segment. Note that the scale of the *x*‐axes is magnified to demonstrate the differences in relative velocities and that the segments have different lengths. Length of the arrows between figures is standardized (i.e., arrows of similar lengths as printed represent similar velocities; the scale is shown in the inset with an arrow denoting 1 mm/s).

To visualize more detailed information about motility, the estimated motion vectors were plotted without averaging over the time dimension. Figure 5 shows a visualization of piecewise aggregates of the velocities only along the centerline dimension, showing the average velocity over time for the beginning, middle and end of each segment.

From these visualizations, the segments could be broadly separated into three identifiable patterns. The first pattern was visible in segments with clear peristaltic motion; this can be identified by the velocity arrows having a dominant direction, signifying intestinal content is being moved along the segment centerline. In the example shown in Fig. 5a, the peristaltic motion was aligned with the defined centerline direction, resulting in positive velocities (i.e., rightward‐pointing arrows). The second pattern was the (near) absence of motility in the segment during the MRI scan. This is characterized by small local velocities across the entire MRI scan, as shown in Fig. 5b. The third pattern was the presence of motility, without peristaltic characteristics. This is characterized either by erratic velocities without any clear coordination, or by waves that push the content back‐and‐forth; an example of the latter option is shown in Fig. 5c. In this figure, segment “a” is from a healthy volunteer; segments “b” and “c” are from Crohn's disease patients. It should be noted that a diseased patient does not necessarily imply a diseased segment, as inflammatory disease does not affect the intestine uniformly. While segment “c” is from a Crohn's disease patient, there is little or no inflammation in this segment.

### Computation Time

The average method runtime was 201 seconds (3 minutes 21 seconds) per segment. The majority of the runtime was used by the registration method, which took an average of 199 seconds (3 minutes 19 seconds) per segment to compute all required deformation vector fields. The remaining operations took 2 seconds in total per segment. The method runtime scales approximately linearly with the number of time points in the dynamic scan, as each additional time point adds two registrations (one for motion estimation, one for centerline propagation).

## Discussion

This study describes a method for quantifying small intestinal motility in 3D cine‐MRI that is capable of automatically detecting, quantifying, and characterizing intestinal motion. To the best of our knowledge, this is the first noninvasive method for small intestinal motility quantification that has this capability. The method yields detailed yet interpretable measurements for motility assessment in the small intestine. This is achieved by employing deformable image registration and evaluating the components of the resulting deformation vector fields that align with the directions of a small‐intestinal centerline. These displacement vectors can be interpreted as the local velocity of the intestinal content. In turn, those velocities can be integrated along the intestinal segment to find the local strength of peristaltic motion. Aggregating the absolute values of the velocities instead results in a metric for the magnitude of motility, irrespective of consistent direction along the intestine and over time. The combination of these metrics could be valuable for the diagnosis of patients with disturbed peristaltic motion, as they do not necessarily suffer from intestinal hypomotility.^26^ Additionally, as the method locally describes the motion parameters, it could be used for disease monitoring, and for identifying intestinal segments that are affected more or less by disease.

While the results of this study demonstrate immediate benefit for the noninvasive assessment of peristalsis from 3D cine‐MRI scans, the research implications are broader than peristaltic movement quantification only. The spatio‐temporal relationship of the velocity vectors along each intestinal segment can reveal distinct patterns in the local intestinal motility. This means these vectors can be used to quantitatively describe the local motion in more detail. Previous research has suggested such motion patterns may be important biomarkers, for example in identifying subgroups of patients with irritable bowel syndrome.^5^ A possible application in future work could be to identify clusters of patients with gastrointestinal diseases and syndromes for which specific patterns are over or underrepresented, which may lead to the discovery of new biomarkers, driving diagnosis and treatment research.

To demonstrate the presented method, the proposed motility metrics were calculated in segments of small intestines for both healthy volunteers and Crohn's disease patients. The motility metrics indicate significantly more active motility in the set of healthy volunteers than in the set of patients, which is consistent with previous studies.^27^, ^28^, ^29^ Additionally, the results showed the proposed motility metrics have a strong discriminative performance both for differentiating motile intestinal segments from non‐motile segments and for detecting the presence and relative direction of peristalsis. Furthermore, we showed that for classifying the presence or absence of motility and propulsive movement, a simple classifier based only on the aggregated motility metrics has a strong agreement with reader consensus labels, outperforming the agreement between two human raters. This suggests that our automatic method can outperform the human inter‐observer variability.

The method developed in this study produces velocity metrics that describe the movement within the small intestinal lumen. Related velocity metrics have previously been proposed for describing esophageal peristalsis^30^ and gastric peristalsis,^31^, ^32^ using manometry and cine‐MRI. It should be noted that the velocities in these previous studies do not describe the exact same quantity: peristaltic velocity as defined in the above two studies refers to the velocity of the peristaltic waves, which is subtly different from the average velocity in the lumen of an intestinal segment. The latter includes the velocity of the intestinal wall at the points of contraction. The velocity component in the direction tangent to the intestinal centerline of the intestinal wall is zero, meaning the average velocity in the intestine is always lower than the velocity of a peristaltic wave. Hence, one should be careful when comparing these quantities to velocities from other methods.

To simplify the evaluation in this study, a large number of intestinal segments were automatically extracted upfront, in order to create a reference set for motion characterization that could be rated by multiple readers. To avoid introducing diverging datasets between different raters, centerline segments that did not pass quality control were immediately discarded. This resulted in a large number of “failed extractions” being excluded from the evaluation. This is not fully representative of the envisioned typical use of the method. When the method is used in practice, centerlines with extraction mistakes could instead be re‐generated using a different starting coordinate, or could be manually cropped to the valid region by the user (depending on the severity of the extraction error). This would result in valid motility metrics being generated for segments that are marked extraction failures when extracted in a non‐interactive setting.

Using mid‐range consumer hardware, the developed method has a runtime of approximately 2 minutes per available time point in the MRI scan, divided by the number of available GPUs. The runtime is almost completely attributed to our chosen registration method, which operates on each time point separately. The chosen method was designed to sacrifice speed for robustness, which was considered more important for this work. This means runtime increases linearly with the number of time points in the scan. While this is not a problem in research settings, a large number of GPUs is (currently) not typically available in clinical settings. Future work could investigate the use of more computationally efficient registration methods.

The goal of this work was to demonstrate a proof‐of‐principle for quantifying small intestinal motility in 3D cine‐MRI. For this purpose, unbiased and representative inclusion was not a concern and hence it was not controlled for. However, it should be noted that as a result, the distributions of the motility scores derived in this work may not be representative of the typical distributions for any specific populations.

### Limitations

First, while the presented method is theoretically robust to the presence of breathing motion in the MRI scans, in this work we only considered scans acquired during breath‐hold. The reason for this is the difficulty of the manual annotation task required for the evaluation in this work. Annotating the segments during breath‐hold resulted in a high level of inter‐rater variability. The presence of breathing motion would make this task even harder, resulting in even less reliable reference annotations. Additionally, due to the dependence on the optical flow in the center of the intestines, our method is not robust to the presence of a (non‐moving) manometric catheter in the intestines. This hampers simultaneous acquisition of both measurements at the same location. While this is not a problem for clinical practice, it prevents us from reliably validating the presented method in a free‐breathing setting. Such validation is currently considered an open problem that we leave to future work.

A second limitation is that the presented method is reliant on the presence of a preparation drink for sufficient contrast of the intestinal lumen. Previous studies have shown that the composition of intestinal content can influence the intestinal motility.^7^, ^26^, ^33^, ^34^, ^35^ The reliance on a preparation drink limits the options for varying this composition and prevents us from studying the completely fasted bowel with the presented method.

Third, the orientation of extracted segments in this work is arbitrary with respect to the physical intestinal tract. The small field of view of the scans in the anterior–posterior direction (35 mm) means it is not possible to determine the true connectivity between separate segments (and, by extension, their orientation with respect to the stomach and the large intestine). Hence, the observed patterns in the intestine motility in isolated segments cannot discern the physical proximal‐distal direction in asymmetric motility patterns, if the segment‐of‐interest does not directly connect to a discernible key point in the field‐of‐view (i.e., the duodenum or the terminal ileum). To obtain directional information, the used MRI motility scan should either include the majority of the intestines, or the scan should be matched to a larger overview scan in which the full connectivity can be evaluated. Knowledge of the full connectivity could additionally enable automatic quality control based on the presence or absence of a single path from the duodenum to the terminal ileum. This would reduce the need for manual quality control of the centerlines.

To our knowledge, a cine‐MRI scan covering the entire abdomen in 3D with high enough framerate to capture small intestinal motility is currently unavailable, meaning only 3D scans with limited field‐of‐view are available for developing quantification methods. Consequently, we can only assess separate segments of the small intestine with cine‐MRI rather than the entire small intestine. An additional limitation stemming from the limited field‐of‐view is the ambiguity that arises at the image borders when peristaltic motion pushes into or out of the image border, or when breathing motion shifts this border along the centerline segment manifold. Our proposed velocity metrics are unreliable near the image boundaries in these scenarios, as the true boundary conditions are unknown.

Finally, it is important to note that this work included only a small number of subjects, and the reference used in this work cannot be considered a ground‐truth. The raters assigned intestinal segments based on visual evaluation and their best judgment on what constituted the presence of motility and the presence of a dominant peristaltic direction. While their judgments correlate, the results show a lot of disagreements between the two raters. The consensus results can be considered a more reliable reading than the results from individual raters, but there is still uncertainty in this reference, especially as the consensus was established by only two raters.

## Conclusion

We have presented a method for quantification of small intestinal motility from noninvasive 3D cine‐MRI scans, which is capable of detailed characterization of motility. This method can be used to quantify motility, to analyze intestinal motion patterns and to detect disturbed peristaltic motion. This can enable the use of detailed noninvasive 3D quantitative analysis of small intestinal motility, both in research and in clinical settings.

## Conflict of Interest

The authors declare no conflicts of interest.

## Supporting information


**Video S1:** An animation of three untangled representations of intestinal segments. The arrows indicate the estimated velocity in the beginning, middle and end parts of each segment.

## APPENDIX A Annotation Protocol

The annotators were asked to manually determine the gradability and class of all segments in the dataset. They were presented with two sets of linked scrollable views from three directions. The right side of the screen showed the static timepoint in which the centerline was extracted and the left side showed a cinematic view of the same patient. The segment to be graded was indicated by numbered markers in both views. The top left of the screen displayed the instructions as shown below at all times. While both raters scored their confidence for the classification of each segment, these ratings were later determined to be too subjective to be useful and were excluded from further analysis.

**Table:**

Instructions	Selection Options
For each segment, indicate whether motile activity is present (either mixing or peristaltic). If so, indicate whether the dominant peristaltic direction is from low to high, from high to low markers, or random/chaotic (i.e., just mixing motion). Assess your confidence from 1 (low confidence) to 5 (high confidence). Markers indicate segments in the timepoint shown in the right window. They are shown in the left window for convenience, but may not be accurate there. Centerline markers sometimes mistakenly cross the intestinal wall into a different segment. If ≥75% of the markers are in one connected segment, score that segment. If not, consider the marker segment ungradable. Determine centerline gradability Select motility present or not Identify peristaltic direction Indicate overall confidence of 2) and 3)	Gradability: Gradable Ungradable (failed centerline) Ungradable (FOV or image quality) Motility: Active motility Motility in <50% of segment No activity Peristaltic direction: Chaotic/random/bidirectional Low to high marker numbers High to low marker number Confidence: 1 (very uncertain) 2 3 4 5 (very certain)
